# Macrophages promote the progression of premalignant mammary lesions to invasive cancer

**DOI:** 10.18632/oncotarget.14913

**Published:** 2017-01-31

**Authors:** Emily C. Carron, Samuel Homra, Jillian Rosenberg, Seth B. Coffelt, Frances Kittrell, Yiqun Zhang, Chad J. Creighton, Suzanne A. Fuqua, Daniel Medina, Heather L. Machado

**Affiliations:** ^1^ Department of Biochemistry and Molecular Biology, Tulane School of Medicine, New Orleans, LA, USA; ^2^ CRUK Beatson Institute and Institute of Cancer Sciences, University of Glasgow, Glasgow, UK; ^3^ Department of Molecular and Cellular Biology, Baylor College of Medicine, Houston, TX, USA; ^4^ Dan L. Duncan Comprehensive Cancer Center Division of Biostatistics, Baylor College of Medicine, Houston, TX, USA; ^5^ Lester and Sue Smith Breast Center, Baylor College of Medicine, Houston, TX, USA

**Keywords:** inflammation, macrophage, early stage lesion, breast cancer progression, mammary

## Abstract

Breast cancer initiation, progression and metastasis rely on a complex interplay between tumor cells and their surrounding microenvironment. Infiltrating immune cells, including macrophages, promote mammary tumor progression and metastasis; however, less is known about the role of macrophages in early stage lesions. In this study, we utilized a transplantable p53-null model of early progression to characterize the immune cell components of early stage lesions. We show that macrophages are recruited to ductal hyperplasias with a high tumor-forming potential where they are differentiated and polarized toward a tumor-promoting phenotype. These macrophages are a unique subset of macrophages, characterized by pro-inflammatory, anti-inflammatory and immunosuppressive factors. Macrophage ablation studies showed that macrophages are required for both early stage progression and primary tumor formation. These studies suggest that therapeutic targeting of tumor-promoting macrophages may not only be an effective strategy to block tumor progression and metastasis, but may also have critical implications for breast cancer prevention.

## INTRODUCTION

Inflammation is a critical component of the tumor microenvironment that is required for tumor progression. While the immune system is a driving force against tumor initiation, anti-tumor immune responses may be subverted to allow for tumor formation and progression. Infiltrating inflammatory cells, including macrophages, promote tumor progression and metastasis in a number of cancers, such as prostate, bladder, colon and breast [1, 2]. Studies aimed at therapeutically targeting tumor-driving components of the immune system are currently in progress and may be key to preventing cancer progression.

The tumor microenvironment plays a pivotal role in promoting breast cancer initiation, progression and metastasis. In particular, tumor-associated macrophages have been shown to promote breast cancer cell invasion and have been correlated with poor prognostic signs such as high tumor grade, low estrogen and progesterone status, high mitotic activity, and metastasis [3]. Although infiltrating macrophages were originally believed to exhibit anti-tumor activity, it is now well-established that once educated by the tumor microenvironment, macrophages secrete a number of factors to promote tumorigenesis. Numerous cell types influence macrophage function including the tumor cells themselves, which not only recruit macrophages to both primary and metastatic tumor sites by secreting chemokines such as CCL2, CCL5 and CXCL12, but also differentiate and polarize them to a tumor-promoting phenotype [4].

In mouse models of breast cancer, macrophages contribute to tumor progression through a variety of mechanisms including IL-10 and arginase 1 (Arg1)-mediated immunosuppression, MMP 7/9 and CCL18-induced matrix remodeling, and EGF and TNFα-stimulated tumor cell migration and metastasis [5–7]. These different functions are carried out by distinct macrophage subpopulations, reflecting the heterogeneity and plasticity that macrophages display in response to local cues. There are several markers that are commonly used to assess the phenotype of macrophages. The mannose receptor CD206, and the macrophage scavenger receptor CD204 are prototypic markers of anti-inflammatory macrophages and their expression negatively correlates with prognosis in numerous types of cancer. CD206 expression has been associated with increased MHCII expression and reduced pro-inflammatory cytokine secretion. Expression of CD204 by tumor-associated macrophages has been linked to suppression of anti-tumor immunity, the clearance of apoptotic cells, and tumor cell invasion and metastasis [8, 9].

Breast cancer is believed to arise in a stepwise fashion, beginning with atypical ductal hyperplasia (ADH), to ductal carcinoma *in situ* (DCIS), to invasive ductal carcinoma (IDC; primary tumor formation), and ultimately progressing to metastatic ductal carcinoma [10]. While it is well-established that macrophages are recruited to the invasive fronts of established tumors to promote angiogenesis and metastasis, fewer studies have focused on the role of macrophages in early stage lesions. One limitation in the field is the lack of *in vivo* models that recapitulate the early stages of ADH/DCIS progression. In this study, we utilized a transplantable p53-null mouse model to study factors that contribute to the progression of early stage mammary lesions [11]. Orthotopic transplantation of p53^-/-^ mammary epithelial cells into wildtype mice led to the development of premalignant lesions that varied in breast cancer subtype, pathobiology, histology, and the ability to progress to invasive cancer. Two of these previously characterized lines, PN1a and PN1b, form estrogen and progesterone receptor positive ductal hyperplasias that are dependent on ovarian hormones for tumor formation and have been maintained by serial passaging in Balb/c mice [12]. Here, we show that macrophages are recruited to premalignant lesions with a high tumor-forming potential, where they are polarized toward a tumor-promoting subtype. In contrast, infiltrating macrophages were rarely found in lesions with a low tumor-forming potential. The immune cell components of the pre-invasive lesions were characterized and suggested that as hyperplasias progress to invasive cancer, numerous anti-inflammatory and immunosuppressive pathways are induced. Macrophage ablation studies showed that macrophages were required for early stage progression and primary tumor formation. These studies demonstrate that in this mouse model, recruited macrophages are instructed to differentiate into a tumor-promoting phenotype, which is crucial for the progression of early stage lesions. Understanding the molecular pathways that recruit and polarize macrophages will have critical implications for therapeutically blocking the tumor-promoting activities of macrophages.

## RESULTS

### Macrophages are recruited to early stage lesions

To investigate mechanisms that drive tumorigenesis in early breast cancer progression, we employed a transplantable model of early progression. As described previously [11], transplantation of p53^-/-^ mammary epithelial cells into the cleared mammary fad pads of syngeneic wildtype mice led to the formation of several premalignant lines that histologically and genetically recapitulated the various subtypes of human breast cancer. Two of these lines, PN1a and PN1b, were derived from contralateral outgrowths from the same mouse, and tissue from these outgrowths was serially transplanted for over 10 generations to confirm stability [11].

**Figure 1:**
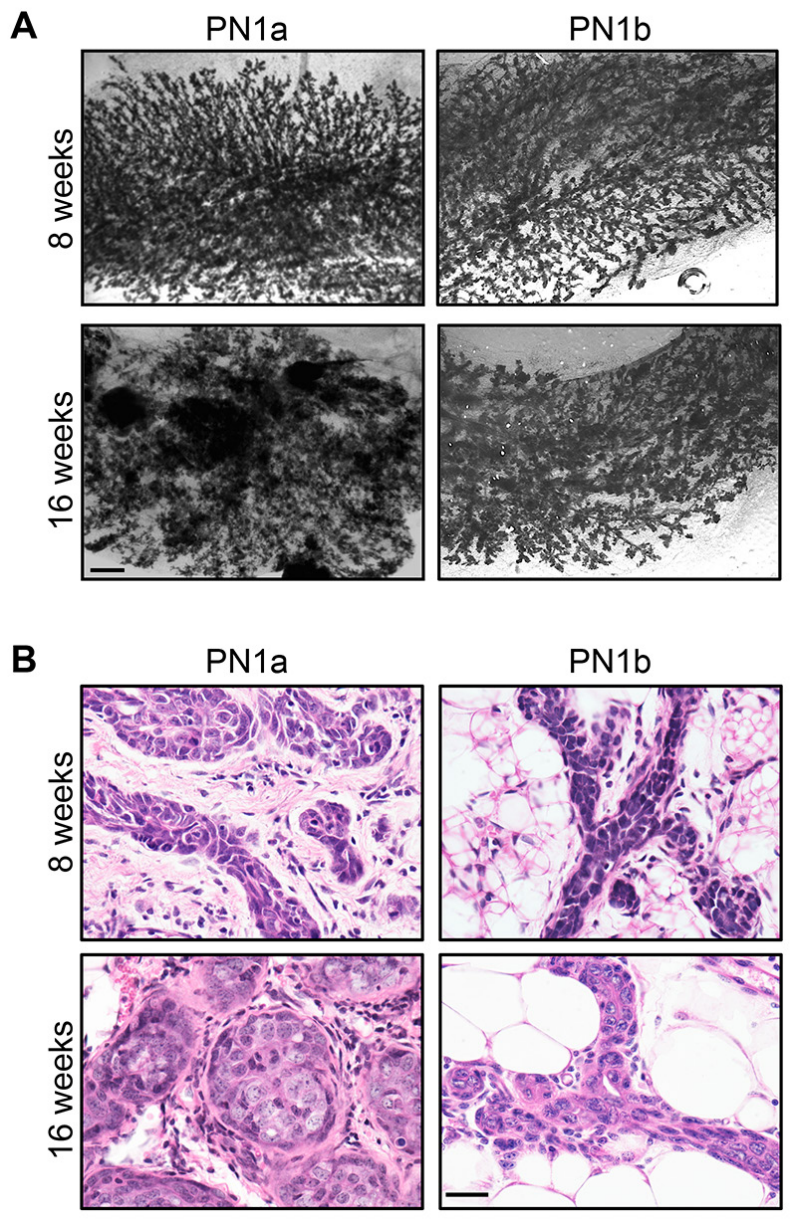
PN1a lesions progress to invasive cancer PN tissue was transplanted into the cleared fat pads of 3 week old Balb/c mice and allowed to grow for 8 or 16 weeks. **A.** Representative whole mount preparations of carmine alum stained mammary glands. Scale bar = 2 mm. **B.** Representative 60X images of H&E staining of PN1a and PN1b lesions at 8 or 16 weeks post-transplantation. Scale bar = 5 μm.

Examination of whole mounted PN1a and PN1b lesions shows that while both lesions exhibit a hyperplastic ductal morphology at 8 weeks post-transplantation, PN1a lesions progress to form nodules by 16 weeks (Figure 1A) [11]. Histological analysis shows that at 8 weeks post-transplantation, both PN1a and PN1b lesions exhibit a well-differentiated ductal hyperplastic morphology. At 16 weeks post-transplantation, PN1a lesions develop a poorly differentiated, lobuloalveolar morphology with multifocal regions of atypical cells in solid nests, while PN1b lesions remain low grade, ductal hyperplasia (Figure 1B). Previous studies demonstrated that PN1a lesions have a high tumor-forming potential resulting in palpable tumors by 24 weeks. In contrast, ~15% of PN1b lesions progress to palpable tumors after 1 year (low tumor-forming potential) [11].

**Figure 2:**
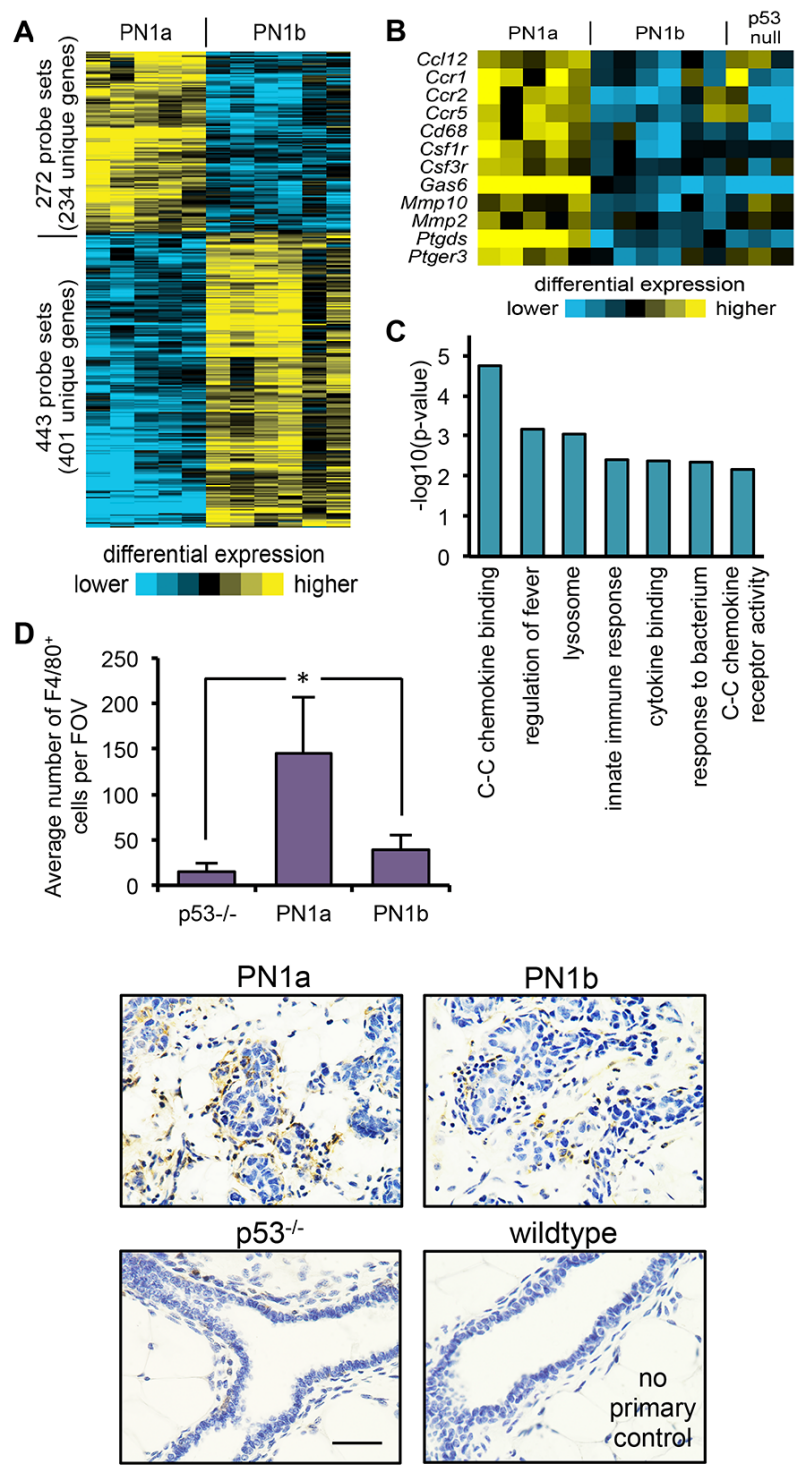
PN1a lesions have increased infiltrating macrophages as compared to PN1b lessions **A.** Heat map depicting gene expression patterns that are altered in PN1a and PN1b lesions at 8 weeks post-transplantation. **B.** Heat map depicting selected immune cell-associated genes that were significantly changed in PN1a lesions as compared to PN1b and p53^-/-^ lesions. **C.** Graphical representation of gene ontology terms significantly increased in PN1a as compared to PN1b lesions. P-values were determined by a one-sided Fisher's exact test. **D.** Top: Graph shows quantitation of the average number of F4/80^+^ cells per field of view (FOV) in p53^-/-^ glands, PN1a and PN1b. For each lesion, a minimum of 5 FOV were captured under 40X and F4/80^+^ cells were counted from 4 lesions (4 mice) for each group. Bottom: Representative 60X images of F4/80 staining in PN1a and PN1b lesions at 8 weeks post-transplantation, and p53^-/-^ mammary glands 8 weeks of age. A no primary antibody control from normal wildtype mammary glands is depicted. Scale bar = 20 μm. *p<0.05.

In order to identify genes important for the transition to invasive cancer, gene expression analysis was performed on PN1a and PN1b lesions at 8 weeks post-transplantation. The results showed that approximately 635 transcripts were differentially expressed (p<0.01 by t-test, fold change >1.5) in PN1a lesions as compared to PN1b (Figure 2A). Gene Ontology analysis revealed the alteration of genes associated with inflammation and innate immunity (Figure 2C). In support, a large cluster of immune cell-associated genes were significantly upregulated in PN1a as compared to PN1b and p53^-/-^ lesions. These transcripts included several macrophage markers such as *Ccr2*, colony stimulating factor 1 receptor (*Csf1r*), *Ccr5, Csf3r, and Cd68* (Figure 2B), suggesting an important role for macrophages in PN1a progression. To confirm theses results, immunohistochemistry was performed using an antibody to F4/80 to detect macrophages. There was a significant increase in infiltrating macrophages in PN1a lesions as compared to PN1b at 8 weeks post-transplantation, demonstrating their recruitment to ductal hyperplasias (Figure 2D). We further examined the macrophage phenotype in PN1a and PN1b lesions by flow cytometry. PN1a lesions were dominated by TIE2^+^CD206^+^ macrophages, a subpopulation known for their pro-angiogenic and immunosuppressive functions [13–15], whereas PN1b lesions also contained a substantial number of TIE2^-^CD206^-^ macrophages. Interestingly, the proportion of CD8^+^ cytotoxic lymphocytes was lower in PN1a as compared to PN1b lesions, consistent with a pro-tumorigenic, immunosuppressive microenvironment (Supplementary Figure 1). These results indicate that recruited macrophages and other immune cells are differentially expressed in pre-invasive lesions with a high tumor-forming potential as compared to lesions that lack invasive potential.

### PN1a-associated macrophages exhibit a tumor-promoting phenotype

**Figure 3:**
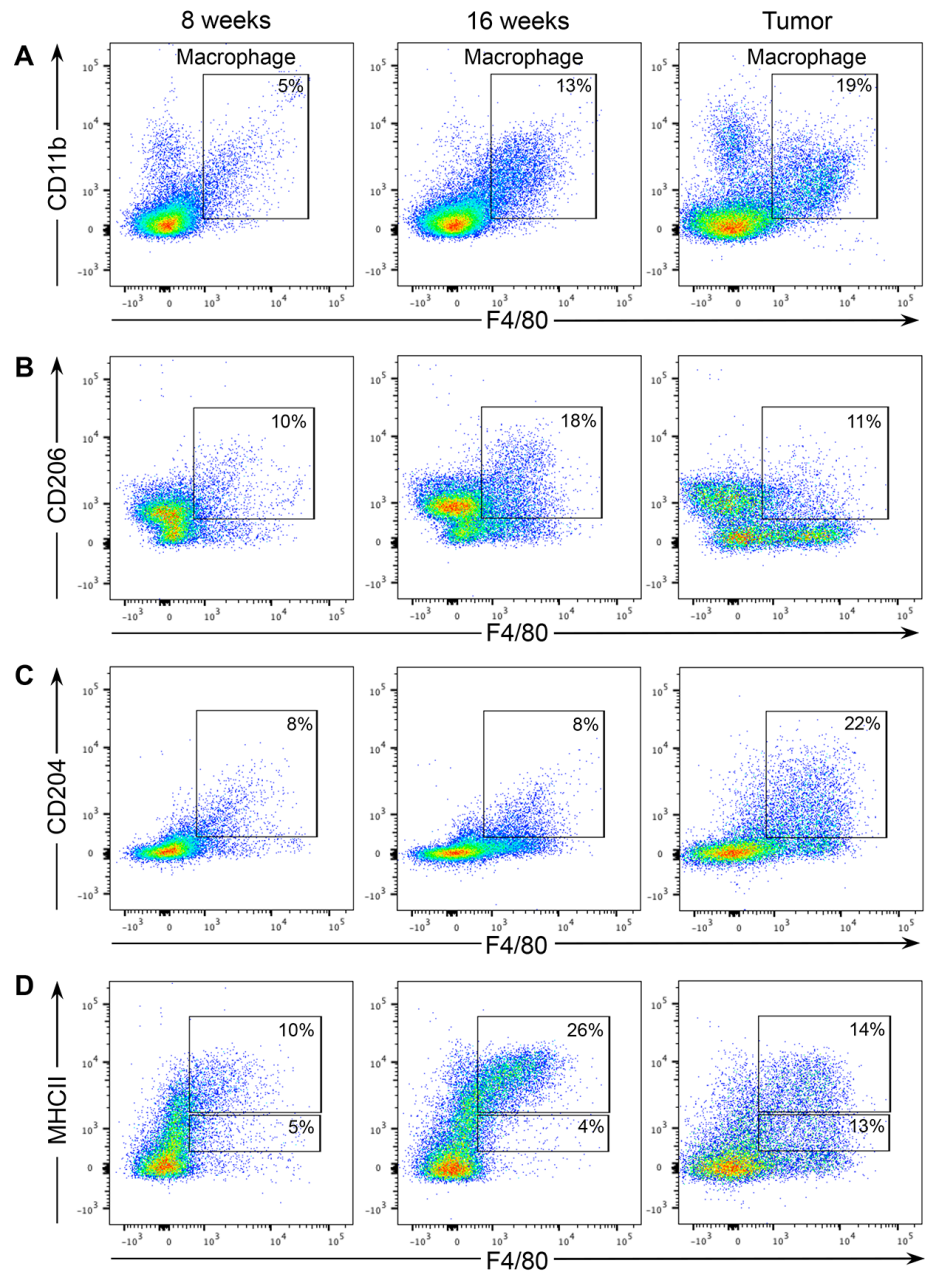
Macrophage populations in early stage PN1a lesions express a mixture of pro- and anti-tumorigenic markers Cells were isolated from PN1a lesions at different stages of progression and analyzed by flow cytometry. For all dot plots, live cells were selected by gating on SYTOX red^-^ cells, and epithelial cells were excluded by gating on CD45^+^ cells. **A.** Dot plots show the numbers of F4/80^+^CD11b^+^ macrophages at different stages of PN1a progression. For **B-D**, CD11b^+^ subpopulations were first selected and further analyzed for the expression of B. CD206 and F4/80, C. CD204 and F4/80 and D. MHCII and F4/80. A minimum of 3 lesions (3 mice) were analyzed for each time point.

Macrophages exhibit an enormous amount of plasticity and can have anti-tumor/pro-inflammatory properties or anti-inflammatory/tumor-promoting activities depending on local microenvironmental cues. Since PN1b lesions contain few recruited macrophages, the remainder of the study focused on characterizing macrophage activity in PN1a lesions exclusively. Flow cytometry was performed on PN1a lesions at various stages of progression to define the macrophage subtype. The percentage of CD45^+^F480^+^CD11b^+^ macrophages increased as PN1a lesions progressed to palpable tumors, suggesting that they may have tumor-promoting capabilities (Figure 3A). After gating on CD45^+^CD11b^+^ subpopulations, we assessed the cell surface expression of the pro-inflammatory macrophage marker, MHCII, and two anti-inflammatory macrophage markers, CD204 and CD206, on F4/80^+^ cells. We observed that CD206 expression peaked at 16 weeks post-transplantation, but decreased in palpable tumors (Figure 3B). In contrast, CD204 expression was low in macrophages in early stage lesions and increased in tumors (Figure 3C). Finally, the percentage of MHCII^HI^ macrophages was higher in early stage lesions, whereas tumors had a higher percentage of MHCII^LO^ macrophages, consistent with CD206 expression (Figure 3D). This is concordant with previous studies that showed that MHCII^HI^ macrophages are increased in early stages of tumorigenesis where they function to recruit other immune cell populations through the production of chemokines such as CCL5, CCL22, and CCL17, and to suppress T-cell proliferation [16]. Together, these results suggest that infiltrating macrophages are dynamic as early stage lesions progress to invasive cancer, and express a spectrum of both pro- and anti-inflammatory markers.

**Figure 4:**
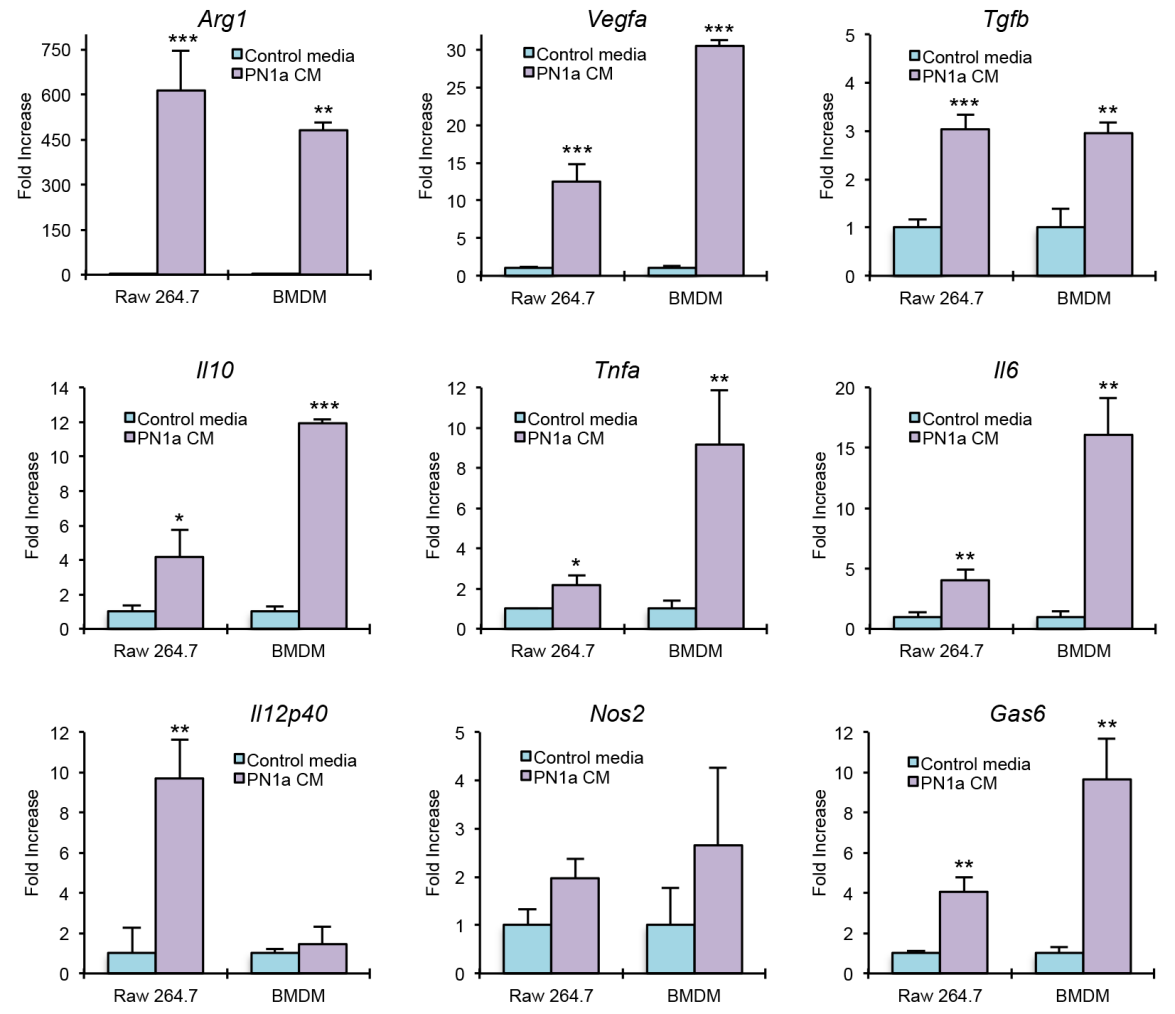
PN1a cells polarize macrophages to a tumor-promoting phenotype BMDMs and RAW 264.7 cells were cultured in the presence of PN1a conditioned media (CM) or control media. After 2 hours, cells were collected and qPCR was performed using primers to *Arg1*, *Il10*, *Vegfa*, *Tgfb*, *Gas6*, *Il12p40*, *Nos2*, *Il6*, and *Tnfa*. Values were normalized to GAPDH or 18S. Values shown are mean and SD (n=3) from one representative experiment. *p<0.05, **p<0.01,***p<0.001.

To determine whether PN1a cells induce macrophage polarization toward a pro-tumor subtype, we exposed the macrophage cell line RAW 264.7 or bone marrow-derived macrophages (BMDMs) to growth media collected from PN1a cells (PN1a conditioned media), or growth media that was cultured in the absence of PN1a cells (control media). After 2 hours of exposure, RNA was isolated from RAW 264.7 cells or BMDMs and various genes were analyzed by qPCR (Figure 4). Several tumor-promoting markers were significantly increased in macrophages cultured with PN1a conditioned media as compared to macrophages exposed to control media. Specifically, *Arg1, Vegfa, Tgfb* and *Gas6* were induced in macrophages exposed to PN1a conditioned media. The pro-inflammatory genes *Il12p40* and *Nos2* (the gene that encodes the enzyme iNOS) were not significantly altered in PN1a-educated BMDMs, although *Il12* was induced in RAW 264.7 cells. *Il10*, which has been shown to be immunosuppressive, was significantly increased in PN1a-educated macrophages. Interestingly, *Il6* and *Tnfa*, which can be both pro-inflammatory and tumor-promoting, were significantly upregulated in macrophages exposed to conditioned media as compared to control media (Figure 4). These results suggest that PN1a cells secrete factors that polarize macrophages toward a tumor-promoting phenotype, and that PN1a-associated macrophages represent a unique subpopulation of macrophages.

**Figure 5:**
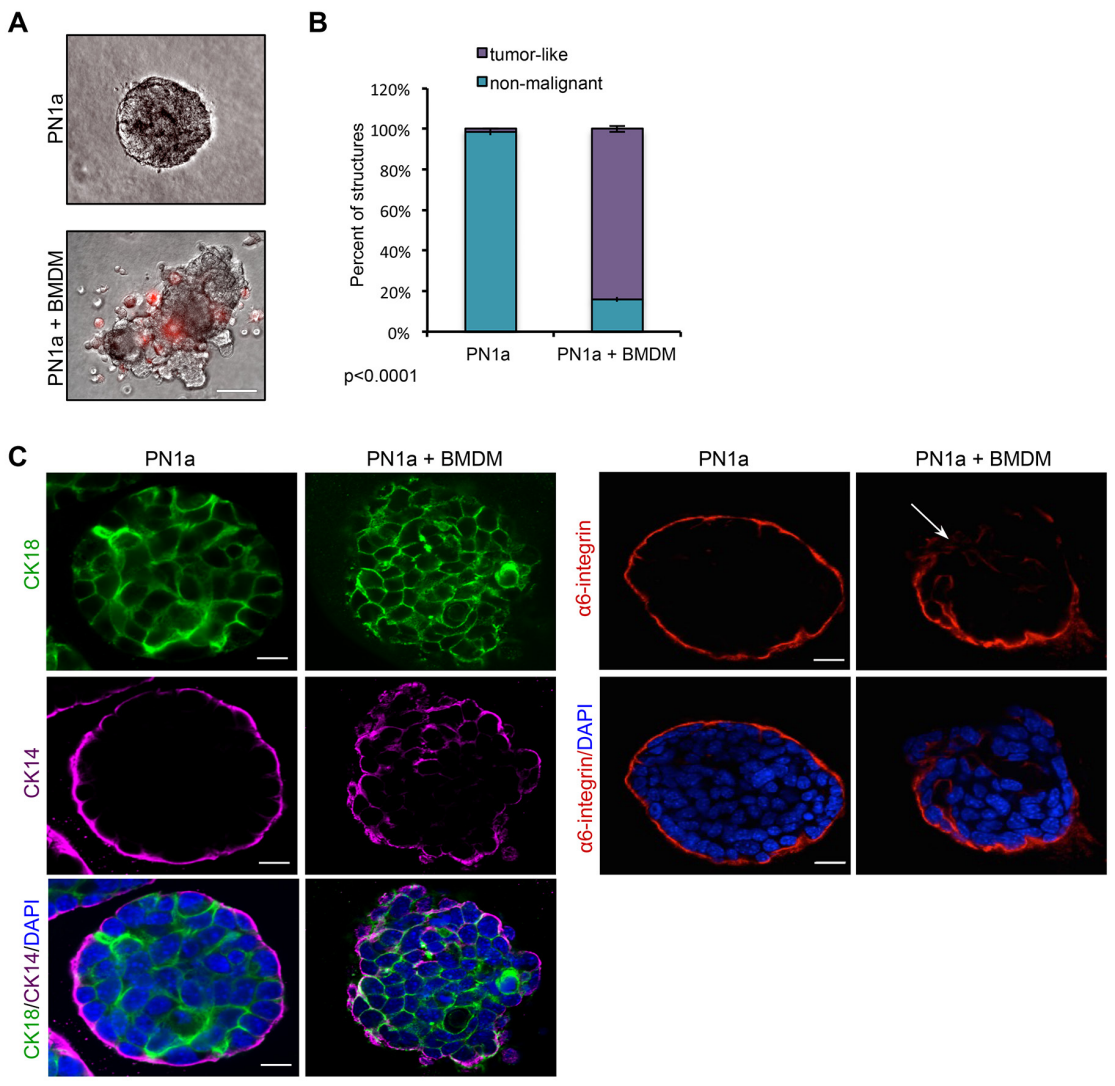
Macrophages induce a malignant phenotype in PN1a cells grown in 3D culture PN1a cells were co-cultured with BMDMs on Matrigel for 10 days. **A.** Representative phase-contrast images of PN1a acini alone (top) or after 10 days of co-culture with PKH26-labelled BMDMs (red) (bottom). Scale bar = 50 μm. **B.** Quantification of the number of tumor-like or non-malignant acini. Approximately 30 acini per group were examined for each experiment from 3 independent experiments. Error bars represent SD, and p-value was determined using a 2×2 contingency table and Chi-squared analysis (p<0.0001). **C.** (Left) Representative confocal images of acini immunostained with antibodies against CK-18 (green), CK-14 (purple), and nuclei were counterstained with DAPI (blue). Scale bar = 25 μm. (Right) Representative confocal images of structures immunostained with antibodies against α6-integrin (red), and nuclei were counterstained with DAPI (blue). Scale bars = 25 μm.

To test whether PN1a-associated macrophages have functional tumorigenic activity, we utilized a 3-D heterotypic culture system. Epithelial cells were isolated from PN1a lesions and cultured in Matrigel as previously described, in which normal mammary epithelial cells form polarized acini with empty lumens and an intact myoepithelium [17]. After 3 days of culture, BMDMs differentiated with L-929 conditioned media, were stained with a fluorescent dye and added to the cultures. After 13 days of culture, PN1a cells in the absence of BMDMs formed uniformly round structures with filled lumens, reminiscent of simple ductal hyperplasia observed *in vivo* (Figure 5A, 5C). Staining with antibodies that mark the basal myoepithelium [cytokeratin (CK) 14 and integrin α6] and the luminal epithelium (CK18) revealed that PN1a cells alone formed an intact basal myoepithelium surrounding hyperplastic luminal cells (Figure 5C). In contrast, co-culture with BMDMs induced a tumor-like phenotype characterized by irregular, disorganized structures and a disrupted basal cell layer. CK14^+^ cells were found throughout the co-cultured structures and integrin α6 was not uniformly expressed at the basal surface, further supporting a malignant phenotype. Quantification of non-malignant and tumor-like structures showed a significant increase in tumor-like PN1a acini in co-cultured cells as compared to PN1a alone (Figure 5B). Notably, BMDMs were recruited to the PN1a cells, suggesting that PN1a cells secrete chemokines that attract the BMDMs. Collectively, these results indicate that PN1a cells recruit and polarize macrophages to a tumor-promoting phenotype.

### Macrophage depletion delays progression to invasive cancer

**Figure 6:**
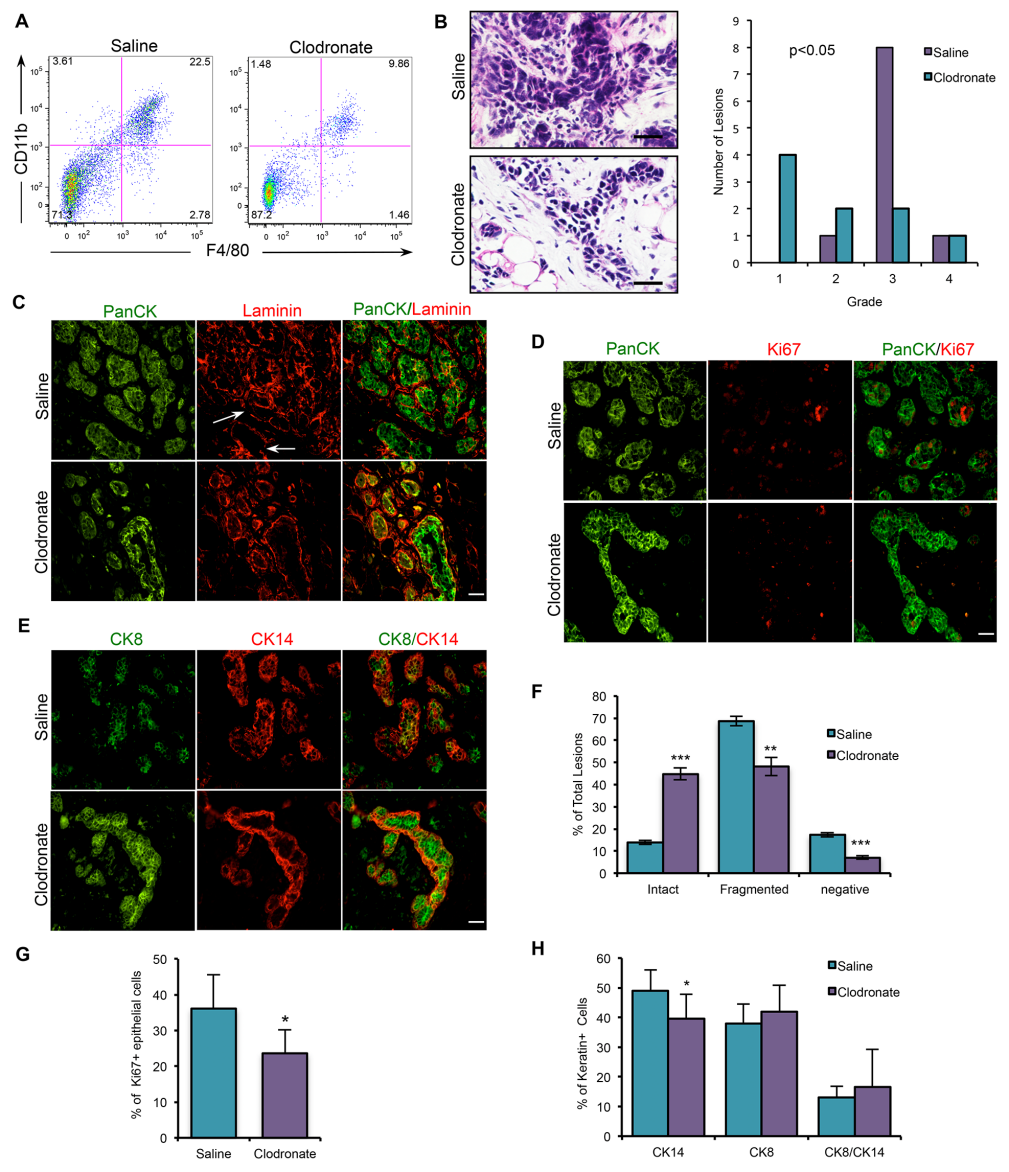
Macrophage depletion using clodronate liposomes impairs the progression of PN1a lesions **A.** Dot plot depicts that the number of CD45^+^CD11b^+^F4/80^+^ cells is decreased in PN1a lesions in clodronate liposome-treated mice as compared to saline-treated. **B.** H&E analysis and quantification of histological grade of saline- and clodronate liposome-treated lesions. Values shown are the number of lesions classified for each histological grade, and a one-way ANOVA with Tukey post-hoc test was used to determine significant differences in grade (p< 0.05). **C-E.** Representative images of immunostaining of early stage PN1a lesions from saline- or clodronate-treated mice using antibodies to C. pan-cytokeratin (PanCK, green) and laminin (red), showing intact or fragmented (white arrow) basement membranes, D. PanCK (green) and Ki67 (red), E. CK8 (green) and CK14 (red). **F.** Quantification of the integrity of the basement membrane as defined by intact, fragmented or negative. A minimum of 5 FOVs were counted for each lesion, and a minimum of 10 lesions (6 mice) were analyzed for each treatment group (n=10). **G.** Graph depicts the percentage of Ki67^+^ proliferating cells, calculated by counting total epithelial cell number by co-staining with PanCK. A minimum of 6 FOVs were counted per each lesion, and a minimum of 6 mice (9 lesions) were counted for each treatment group. **H.** Quantification of CK8 and CK14 staining. Ten FOV were counted for each lesion and a minimum of 10 lesions (6 mice) were counted for each group. Values are shown as the mean + SD. *p<0.05, **p<0.01, ***p<0.001. Scale bars = 20 μm.

To test whether macrophages have tumor-promoting activity *in vivo*, macrophages were depleted in PN1a-bearing mice using clodronate liposomes. Equal numbers of PN1a cells were transplanted into the cleared fat pads of Balb/c mice and allowed to grow for 2 weeks. Then, macrophages were depleted by i.v. administration of clodronate liposomes every 3 days for an additional 4 weeks. Flow cytometry showed a ~2 fold depletion of macrophages in clodronate liposome-treated animals in both the bone marrow (Supplementary Figure 2A) and in PN1a lesions (Figure 6A) as compared to saline-treated animals, which is consistent with previous reports [18]. Immunostaining verified that macrophages were significantly depleted in areas adjacent to the lesions (Supplementary Figure 2D), and carmine-stained whole mount analysis showed that there was no significant difference in the percent of fat pad filled between saline- and clodronate-treated PN1a lesions (Supplementary Figure 2B), indicating that macrophage depletion did not effect the outgrowth potential of PN1a lesions. To address whether macrophages are required for early stage progression, the consequences of macrophage depletion was first addressed by determining histological grade of the pre-invasive lesions, as depicted in Supplementary Figure 2B. While 80% of the control lesions were classified as Grade 3, the majority of the macrophage-depleted lesions were Grade 1/2, suggesting a significant delay in early progression (Figure 6B). The integrity of the basement membrane surrounding the PN1a epithelium was examined by immunostaining for the basement membrane protein laminin. Our data show that there was a significant increase in the number of lesions containing an intact basement membrane in macrophage-depleted lesions as compared to macrophage-containing lesions (Figure 6C, 6F). In support, the number of proliferating cells was significantly decreased in macrophage-depleted lesions as compared to controls (Figure 6D, 6G). Finally, immunostaining using antibodies to CK14 and CK8 (luminal) showed a significant decrease in basal cells in clodronate liposome-treated animals as compared to control mice, consistent with well-differentiated, low grade lesions (Figure 6E, 6H). These results suggest that macrophage depletion delays the progression of pre-invasive lesions during the early stages of tumorigenesis.

**Figure 7:**
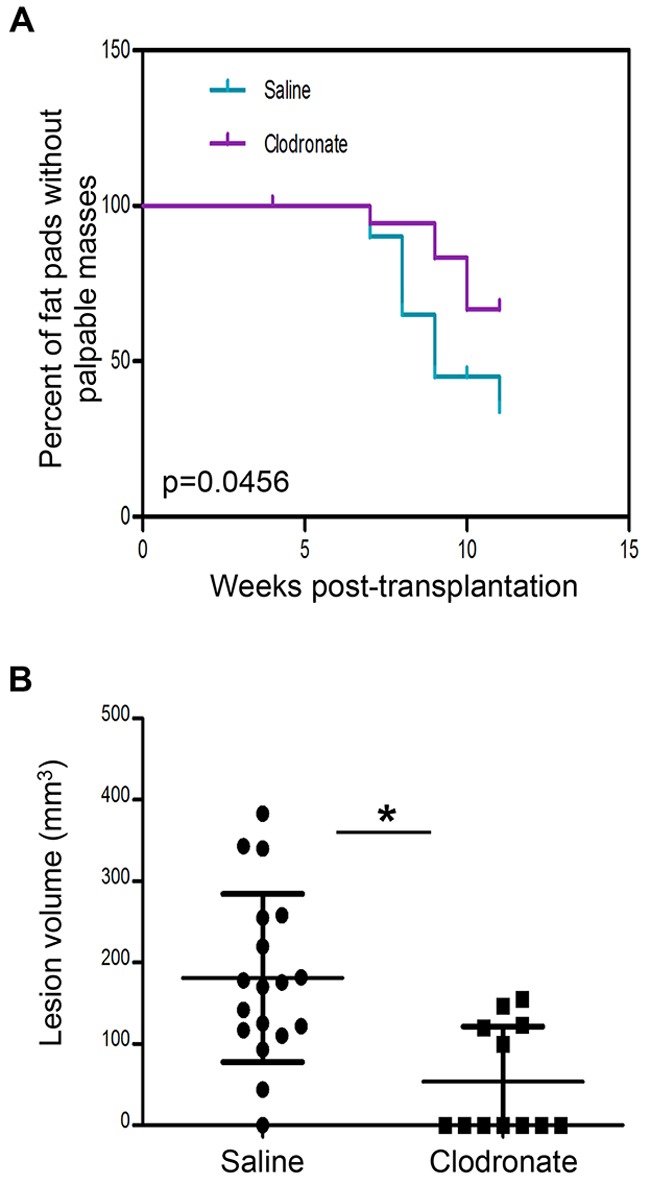
Macrophage depletion using clodronate liposomes impairs PN1a tumor formation *in vivo* PN1a-bearing mice were administered clodronate liposomes (or saline control) every 3 days for 9 weeks (11 weeks post-transplantation), to allow for palpable tumor formation. **A.** Kaplan-Meier curve showing the number of fat pads with palpable masses at 0-11 weeks post-transplantation. The log rank test was used for statistical analysis (p=0.0456), and 20 lesions (10 mice) were included for each group. **B.** Scatter plots show quantification of tumor volume (mm^3^) at 11 weeks post-transplantation for saline-treated (18 lesions/tumors, 10 mice) or clodronate liposome-treated (12 lesions/tumors, 11 mice) *p<0.05.

Next, we asked whether macrophages were required for primary tumor formation. For these experiments, clodronate liposomes were administered every 3 days for 9 weeks (11 weeks post-transplantation), to allow for palpable tumor formation. Mammary tumors began to form as early as 7 weeks post-transplantation in both groups, however by 11 weeks, there was a significant reduction in palpable tumors in macrophage-depleted mice as compared to saline-treated animals. Furthermore, there was a significant decrease in tumor volume at 11 weeks post-transplantation in macrophage-depleted mice as compared to control mice (Figure 7). Collectively, these results suggest that macrophages in early stage lesions are tumor-promoting and that depletion of macrophages in pre-invasive lesions significantly delays primary tumor formation.

## DISCUSSION

Early mammary lesions such as ADH and DCIS confer an increased relative risk for the development of breast cancer later in life [19]. Currently, the mechanisms that mediate the transition from pre-invasive lesions to invasive breast cancer are largely unknown. In this study, we utilized a unique transplantable model of early progression to identify factors driving the progression of premalignant mammary lesions. Gene profiling showed increased expression of several inflammatory chemokines and macrophage markers in PN1a lesions, which have a high tumor-forming potential, as compared to PN1b lesions, which rarely progress to tumors (Figure 2). Immunohistochemistry and flow cytometry showed that macrophages were recruited to ductal hyperplasias in PN1a lesions where they expressed cell surface markers characteristic of a tumor-promoting phenotype (Figures 2 and 3, Supplementary Figure 1). In support, macrophages were recruited to PN1a acini in a 3-D co-culture system, where they induced a malignant phenotype (Figure 5). Finally, depletion of macrophages *in vivo* significantly delayed the progression of PN1a lesions to palpable tumors, suggesting a critical role for macrophages in early progression.

Tumor-associated macrophages in established tumors have been shown to promote breast cancer cell invasion, angiogenesis and metastasis [3, 20]. However, their function in early stage lesions has been debated. In the polyoma middle T antigen mouse model of mammary tumorigenesis (MMTV-PyMT), genetic ablation of *Csf1* - a cytokine required for monocyte to macrophage differentiation – resulted in the attenuation of tumor metastasis and a reduction in multiple foci on the distal duct; however, the growth rate and incidence of primary tumors remained unchanged, suggesting that macrophages do not mediate tumor formation [21]. This data stands in contrast to a growing body of literature suggesting that macrophages at the pre-invasive stage have tumor-promoting activities, including immunosuppression and promotion of cell invasion. Schwertfeger and colleagues showed that macrophages are recruited to mammary hyperplasias in response to FGFR activation, where they promote tumor cell migration and invasion in a CXCR2-dependent manner [22, 23]. In a model of ulcerative colitis-induced carcinogenesis, recruited macrophages were shown to have tumor-promoting function and to express numerous pro- and anti-inflammatory cytokines [24]. Likewise, our data suggests that recruited macrophages in ductal hyperplasias exert pro-tumorigenic activities that promote primary tumor formation. This model is highly relevant to human premalignancy as mutations in p53 occur in some cases of ADH as well as 20-40% cases of DCIS [25]. Furthermore, p53-null pre-invasive lesions recapitulate the intra-tumoral heterogeneity displayed in human disease [11]. Macrophage activity during tumor initiation and early stage progression may depend on lesion histopathology, as MRI studies have shown that the infiltration of CD68^+^ cells is strongly associated with high grade comedo DCIS lesions [26]. Moreover, gene expression analysis of patients with DCIS demonstrated that a macrophage response signature is associated with high grade, ER/PR-negative DCIS, and that this signature is conserved during progression to invasive cancer [27]. Thus, the discordant findings on the role of macrophages in early stage mammary lesions may be a reflection of tumor heterogeneity, subtype specificity, or inherent differences of mouse models.

Once infiltrated into the tumor microenvironment, macrophages differentiate and respond to local cues to exert a variety of activities, including immunosuppression, angiogenesis, metastasis, tumor cell survival, migration and invasion [4]. Our *in vitro* data shows that PN1a-secreted factors can induce macrophages to express a unique set of cytokines, including *Arg1* and *Tgfb* (Figure 4), which have been shown to suppress tumor cell proliferation and CD8^+^ T-cell activity [28]. Interestingly, *Il6* and *Tnf* were also induced. While these cytokines have classic roles in inflammation, they have more recently been shown to promote angiogenesis, metastasis and stem cell self-renewal [4, 29]. Finally, the pro-inflammatory cytokine *Gas6* was induced (Figure 4), which has been shown to promote tumor cell survival and invasion [30]. These results suggest that PN1a pre-invasive cells differentiate macrophages toward a pro-tumorigenic phenotype. In support of this notion, others have shown that exposure of CD14^+^ peripheral blood mononuclear cells to breast cancer cell line-secreted factors increases their expression of the scavenger receptor CD163 and the OX-2 membrane glycoprotein, CD200R, while concomitantly decreasing their expression of the T-cell co-stimulatory molecule, CD86 [31]. Our flow cytometry analysis of macrophages in 8 and 16 week PN1a lesions showed high expression of the scavenger receptor, CD206, and MHCII as compared to macrophages in PN1a tumors, which had higher expression of the mannose receptor CD204 and were MHCII^LO^. This increase in MHCII^LO^ macrophages in PN1a tumors is not surprising as MHCII^LO^ macrophages have been associated with a hypoxic tumor microenvironment in breast, lung, and ovarian mouse tumor models [16, 28, 32]. In contrast, MHCII^HI^ macrophages have been shown to be associated with normoxic regions, exhibit a pro-inflammatory, anti-tumor phenotype, and have iNOS-dependent T-cell suppressor activity [16]. The expression of CD206^+^ and MHCII^HI^ populations in PN1a lesions suggest that these macrophages may exert both pro-tumor and anti-tumor functions.

In the developing mammary gland, macrophages are required for normal branching morphogenesis, ductal elongation, and the repopulation ability of mammary stem cells [33, 34]. Emerging evidence indicates that tumor-promoting macrophages support cancer stem cell renewal, maintenance, and migratory capacity through the secretion of numerous factors including IL-6 and TNFα. Although it is unknown how the increase in macrophage infiltration may effect stem cell populations in PN1a lesions, the increased secretion of IL-6 and TNFα may support cancer stem cell function in PN1a lesions, which could contribute to the enhanced tumor-forming potential of PN1a lesions as compared to PN1b lesions. Further studies are required to address this mechanism.

Macrophages are highly plastic, and their phenotype can change in response to a variety of cues from their microenvironment. For instance, tumor and T-regulatory cell-derived IL-10 can skew macrophages toward an anti-inflammatory phenotype characterized by activation of STAT3 and increased expression of CD163, CCL18, and SOCS3. In addition, the cytokine, TNFα, along with IFN-γ induces macrophage expression of the immune-inhibitory receptor, PD-L1, leading to suppressed T-cell proliferation and cytokine production [35, 36]. Although it is unclear which PN1a-derived factors are driving this change in polarization, our microarray showed that prostaglandin was increased in PN1a lesions, which has previously been shown to be a tumor-derived factor capable of polarizing macrophages toward a tumor-promoting phenotype [37]. We also found increased expression of the macrophage chemotactic proteins, CCL2 and CSF-1. Both of these genes have been correlated with poor prognosis in breast cancer patients [38–40]. Moreover, several studies have shown a positive correlation between the number of infiltrating macrophages in invasive ductal carcinoma and the expression of CCL2, CSF-1, and CCL5 [41, 42]. However, *in vivo*, the relative contribution of PN1a cell-derived factors toward macrophage polarization is likely more complex, as the presence of other immune cells such as B lymphocytes and T-regulatory cells may influence macrophage recruitment and polarization.

Over the last decade, considerable progress has been made characterizing tumor-associated macrophages in breast cancer. As a result, there are currently several anti-macrophage therapeutic strategies that have been developed and are currently in clinical trials for the treatment of metastatic breast cancer (clinicaltrials.gov). These include blocking macrophages by inhibiting CSF-1/CSF1R signaling, as well as disrupting cytokines/chemokines that recruit and polarize macrophages to a pro-tumor phenotype. Our studies suggest that preferentially targeting macrophages and/or disrupting macrophage polarization may also be beneficial in patients with early stage lesions. However, further studies are required to understand the mechanisms that drive macrophage recruitment, polarization and function in pre-invasive mammary lesions.

## MATERIALS AND METHODS

### Mice and cell lines

Mice were maintained in a pathogen-free facility in accordance with the NIH Guide for the Care and Use of Experimental Animals with approval from the Tulane School of Medicine Institutional Animal Care and Use Committee. Balb/c mice were obtained from Envigo, and *Trp53*^-/-^ mice (Balb/c) have been described [43]. RAW 264.7 cells (ATCC) were maintained in DMEM containing 10% fetal bovine serum (FBS) (Life Technologies) and 1% penicillin-streptomycin (Life Technologies). L929 cells were a kind gift from Dr. Kathryn L. Schwertfeger's lab (University of Minnesota) and L929 conditioned media was prepared from them as previously described [44]. All cell lines used in these studies were authenticated by STR Profiling (DDC Medical). All tissue culture materials used for the isolation and analysis of macrophages were made of polypropylene.

### Transplantation, whole mount analysis, and immunostaining of mammary glands

PN1a and PN1b lesions were derived from *Trp53*^-/-^ mice (Balb/c) as previously described [11]. For maintaining and expanding tissue, contralateral mammary glands containing PN1a or PN1b lesions after 8 weeks of outgrowth were minced into 1 mm fractions with a scalpel and re-transplanted into the cleared fat pads (#4 contralateral glands) of 3 week old female Balb/c mice (weight 10-13 g) as previously described [45]. For whole mount analysis, mammary glands were fixed in cold 4% paraformaldehyde (Thermo Fisher Scientific) for 2 hours and stained with carmine alum (Sigma Aldrich) overnight. The next day, glands were dehydrated in a series of ethanols and placed in xylene before imaging on a Leica M165 FC stereoscope (Leica Biosystems). Mammary glands were then embedded in paraffin and sectioned, and sections were deparaffinized and rehydrated before staining with hematoxylin and eosin (H&E).

Immunostaining was performed as previously described [46] with the following modifications: immunohistochemistry with F4/80 was performed in absence of antigen retrieval, endogenous peroxidases were blocked by incubating sections in a solution containing 3% hydrogen peroxide and methanol, and M.O.M. blocking reagent (Vector Labs) was used for mouse monoclonal antibodies. Primary antibody incubations were performed according to the manufacturer's instructions (Supplementary Table 1). For each mammary gland (10 mammary glands per antibody), 8-10 random fields of view (FOV) were captured using a Nikon Eclipse Ci microscope (Nikon Instruments) at 40X magnification unless otherwise specified, and positive cells were counted using the NIS-Elements Basic research Software (Nikon Instruments). For quantification of macrophages, F4/80^+^ cells were expressed as the average number of positive cells per FOV. For quantification of Ki67, the number of positive cells was calculated as a percentage of total epithelial cells using panCK co-staining as a marker of epithelium. Laminin expression was expressed as the percentage of lesions counted and lesions were categorized as having an intact, discontinuous, or negative basement membrane status based on previous studies [47].

### Gene expression profiling

Mammary glands from PN1a (n=5) or PN1b (n=6) lesions at 8 weeks post-transplantation, or p53^-/-^ mice (n=3) at 10 weeks of age were removed and homogenized in TRIzol reagent (Life Technologies) to fully lyse the tissue. Total RNA was isolated according to the manufacturer's instructions, followed by Qiagen RNeasy column purification (Qiagen). cRNA was synthesized and hybridized onto Affymetrix MG_U74Av2 chips using recommended procedures for synthesis, hybridization, washing, and staining with streptavidin-phycoerythrin. CEL files were processed using dChip (using PM-MM model and quantile normalization). Differences were defined between PN1a and PN1b samples using a two-sided t-test (on log-transformed values) and fold change. Differences between PN1a and p53^-/-^ samples, and PN1b and p53^-/-^ samples, as well as a complete list of the top differential genes are included in Supplementary Table 2. Gene Ontology term enrichment was determined using the SigTerms tool [48]. Array data were deposited in the Gene Expression Omnibus (accession number GSE84828).

### Flow cytometry

For analysis of immune cells, PN1a glands were dissected from Balb/c mice, minced and digested for 20 minutes with shaking in a DMEM/F12 (Life Technologies) media containing 1 mg/ml of collagenase A (Roche), 25 μg/ml of DNAse I (Roche), and 1% antibiotic-antimycotic (Life Technologies). Lymph nodes were removed from all mammary glands prior to mincing. Digestion was neutralized by adding DMEM/F12 (Life Technologies), supplemented with 10% FBS (Life Technologies), and cells were filtered four times through a 70 μm strainer (BD Bioscience). Cells were then incubated at 4°C with 1 μg/million cells of CD16/CD32 Fc block (BD Bioscience) for 10 minutes and incubated on ice for 30 minutes with fluorophore conjugated anti-mouse antibodies as indicated in Supplementary Table 1. Subsequently, cells were washed twice with Hank's balanced salt solution (HBSS) (Life Technologies) containing 2% FBS (Life Technologies), filtered through a 35 μm cell strainer (BD Bioscience), and stained with 5 nM SYTOX red or 7AAD (Life Technologies). Flow cytometry was performed using a LSRII or FACSAria IIu cell sorter (BD Biosciences) and analyzed with FlowJo v10.1 (Tree Star, Inc).

### Epithelial cell isolation from PN1a lesions

To isolate epithelial cells from PN1a lesions, mammary glands containing 8 week outgrowths of PN1a lesions were minced into 1 mm fractions using a scalpel and digested for 1 hour with shaking in DMEM/F12 (Life Technologies) media containing 1X antibiotic-antimycotic (Life Technologies), 2 mg/ml of collagenase A (Roche), and 25 ug/ml of DNAse I (Roche). Then, the cells were washed with DMEM/F12 (Life Technologies) containing 5% FBS (Life Technologies) and enriched for epithelial organoids by a series of four 3 second pulses at 450 x g [49]. Enriched organoids were trypsinized (0.05% trypsin) for 20 minutes, passed through a 70 μm filter (BD Bioscience), and washed with PBS (Life Technologies). Subsequently, PN1a cells were centrifuged at 600 x g for 5 minutes, trypsinized for 5 minutes, and passed through a 70 μm filter (BD Bioscience). The resulting enriched population was centrifuged for 10 minutes at 600 x g and resuspended in DMEM/F12 media containing 5% FBS (Life Technologies), 1% antibiotic-antimycotic (Life Technologies), 5 μg/ml of insulin (Sigma Aldrich), and 5 ng/ml of epidermal growth factor (EGF) (Millipore) and prepared for experiments.

### Preparation of bone marrow-derived macrophages

Bone marrow was obtained from the femur and tibia of C57BL/6 mice as described [50]. Cells were cultured in a 10 cm petri dish for one week in DMEM (Life Technologies) supplemented with 10% FBS (Life Technologies), 1% penicillin and streptomycin (Life Technologies), and 20% L929 conditioned media. After 7 days of culture, BMDMs were enriched for F4/80-positive cells using the Magnisort F4/80 positive selection kit (eBioscience) according to the manufacturers instructions.

### Two-dimensional co-culture

PN1a cells were isolated from mammary glands containing 8 week old outgrowths as described above. One million cells were seeded in a 6 well plate in DMEM/F12 containing 5% FBS (Life Technologies), 1% antibiotic-antimycotic (Life Technologies), 5 μg/ml of insulin (Sigma Aldrich), and 5 ng/ml of EGF (Millipore), and incubated at 37°C/5% CO_2_. For control media, DMEM/F12 containing 5% FBS (Life Technologies), 1% antibiotic-antimycotic (Life Technologies) was added to a 6 well plate and incubated at 37°C/5% CO_2_. After 72 hours, conditioned or control media was collected and added to BMDMs or RAW 264.7 cells that had been serum starved overnight. After 2 hours, cells were collected in TRIzol (Life Technologies) for gene expression analysis.

### Quantitative RT-PCR

Total RNA was extracted from RAW 264.7 cells or primary BMDMs using TRIzol and DNA-free DNA Removal Kit (Life Technologies) according to the manufacturer's instructions. cDNA was prepared using the iScript cDNA Synthesis Kit (Biorad) and qPCR was performed using SYBR green methodology on a CFX96 Touch Real-Time Detection System (Biorad). The primer sequences are listed in Supplementary Table 3, and the relative gene expression changes were determined using the 2^-ΔΔCt^ method, where GAPDH (RAW 264.7) or 18S (BMDMs) served as the internal control. For each gene, 3 (RAW 264.7 cells) or 4 (BMDMs) biological replicates were analyzed.

### Three-dimensional co-culture

Epithelial cells were isolated from PN1a lesions at 8 weeks post-transplantation as described above and further enriched using EasySep Mouse Epithelial Cell Enrichment Kit (STEMCELL Technologies). Ten thousand PN1a cells were seeded on growth factor-reduced Matrigel® (BD Bioscience) in a Nunc 8 well chamber system (Thermo Fisher Scientific) as described previously [17]. After 3 days, BMDMs that had been isolated as described above were labeled with PKH26 (Sigma Aldrich) and added to the culture. After 10 days of co-culture, the structures were imaged under a 40X objective using an Evos FL microscope (Thermo Fisher Scientific). Subsequently, the slides were fixed with 2% paraformaldehyde, permeablized with 0.5% Triton X-100 (Thermo Fisher Scientific), and washed with PBS containing 100 mM glycine (Thermo Fisher Scientific). Afterwards, the slides were blocked with PBS containing 10% goat serum (Life Technologies), 0.1% BSA (Sigma Aldrich,), 0.2% Triton-X 100 (Thermo Fisher Scientific), and 0.05% Tween-20 (Sigma Aldrich) for 1 hour, and stained with primary antibodies listed in Supplementary Table 1 overnight at room temperature. The following day, slides were washed with PBS containing 10% goat serum (Life Technologies), 0.1% BSA (Sigma Aldrich), 0.2% Triton-X 100 (Thermo Fisher Scientific), and 0.05% Tween-20 (Sigma Aldrich) and stained with anti-mouse or anti-rabbit secondary antibodies conjugated to Alexa Fluor dyes for one hour. Afterwards, slides were wash and mounted withVectashield Mounting medium with 4’,6-Diamidino-2-phenylindole (DAPI) (Vector Labs). Confocal images were acquired under a 60X object using a Nikon A1plus confocal microscope with PMT and GaASP detectors.

### Macrophage depletion

PN1a cells were isolated from mammary glands containing PN1a lesions 8 weeks post-transplantation as described above. Viable cells were counted on a hemacytometer using trypan blue exclusion dye and resuspended at a concentration of 1,000 cells/10 μl in a 1:1 solution of PBS and growth factor-reduced Matrigel® (BD Biosciences). Ten microliters of cells were injected into contralateral cleared fad pads of 3 week old syngeneic mice using a 26 gauge needle and a 50 μl Hamilton glass syringe (Hamilton Company). After two weeks, mice were randomly distributed into groups and began intravenous injection (tail vein) of 100 μl (5 mg) per 10 g of body weight of clodronate-liposomes (clodLIP B.V.) or an equal volume of saline every 3 days. Long-term intravenous administration of clodronate-liposomes has been previously shown to deplete all macrophage subsets in the liver, spleen, and bone marrow as well as precursor blood monocytes in circulation [51]. Different cohorts were treated for the indicated times depending on the experiment. For tumor latency studies, mice were palpated and tumors size was measured with calipers every other day. Tumor volume was calculated at time of sacrifice (11 weeks) based on the formula (LxW^2^)/2. One mouse in the saline group was sacrificed early due to a tumor volume greater than 10% of body weight, and two mice in the clodronate group were sacrificed early do injection site related illness. All 3 mice were censored in the Kaplan-Meier plot, and excluded from tumor volume quantification. Body mass was measured at week 11 to verify health status of all mice included in tumor latency studies.

### Statistical analysis

All tests were performed using GraphPad Prism 5. The significance of fold changes for qPCR and immunostaining results was calculated using an unpaired t-test. Data was verified as Gaussian using the D’Agostino-Pearson Omnibus. For the quantification of histological grade, significance was calculated using one way ANOVA with Tukey post-hoc test. For tumor latency experiments, time was counted from the day of PN1a transplantation until the time of sacrifice. The significance was calculated using the log rank test. For 3-D co-culture experiments, the number of non-malignant and tumor-like acini were counted in each well and the significance was calculated using a 2×2 contigency and Chi-squared analysis.

## SUPPLEMENTARY MATERIALS FIGURES AND TABLES




