# Supplementary material for: Macrophages promote the progression of premalignant mammary lesions to invasive cancer
Source: Oncotarget. 2017 Jan 31;8(31):50731–46. doi: 10.18632/oncotarget.14913 (PMC5584199; doi:10.18632/oncotarget.14913)
Supplement: Supplementary file 1 [file oncotarget-08-50731-s001.pdf]

Macrophages promote the progression of premalignant mammary lesions to invasive cancer

SUPPLEMENTARY MATERIALS

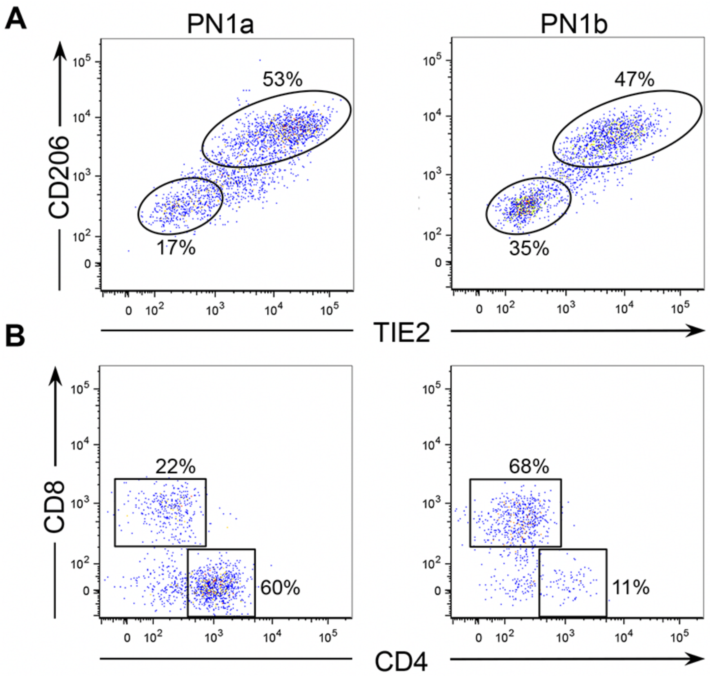

Supplementary Figure 1: Myeloid and lymphoid populations in PN1a and PN1b lesions. A. Dot plots show CD206 and TIE2-expressing macrophages after gating on $CD45^{+}CD11b^{+}F4/80^{+}$ cells in PN1a and PN1b lesions at 16 weeks post-transplantation. **B.** Dot plots depict the proportion of $CD8^{+}$ (cytotoxic T cells) and $CD4^{+}$ (T helper cells) lymphocytes after gating on $CD45^{+}CD11b^{-}CD3^{+}$ cells in PN1a and PN1b lesions at 16 weeks post-transplantation. Data shown represent 1 experiment using 2 pooled lesions (2 mice) in each group, and the experiments were repeated 2-4 times.

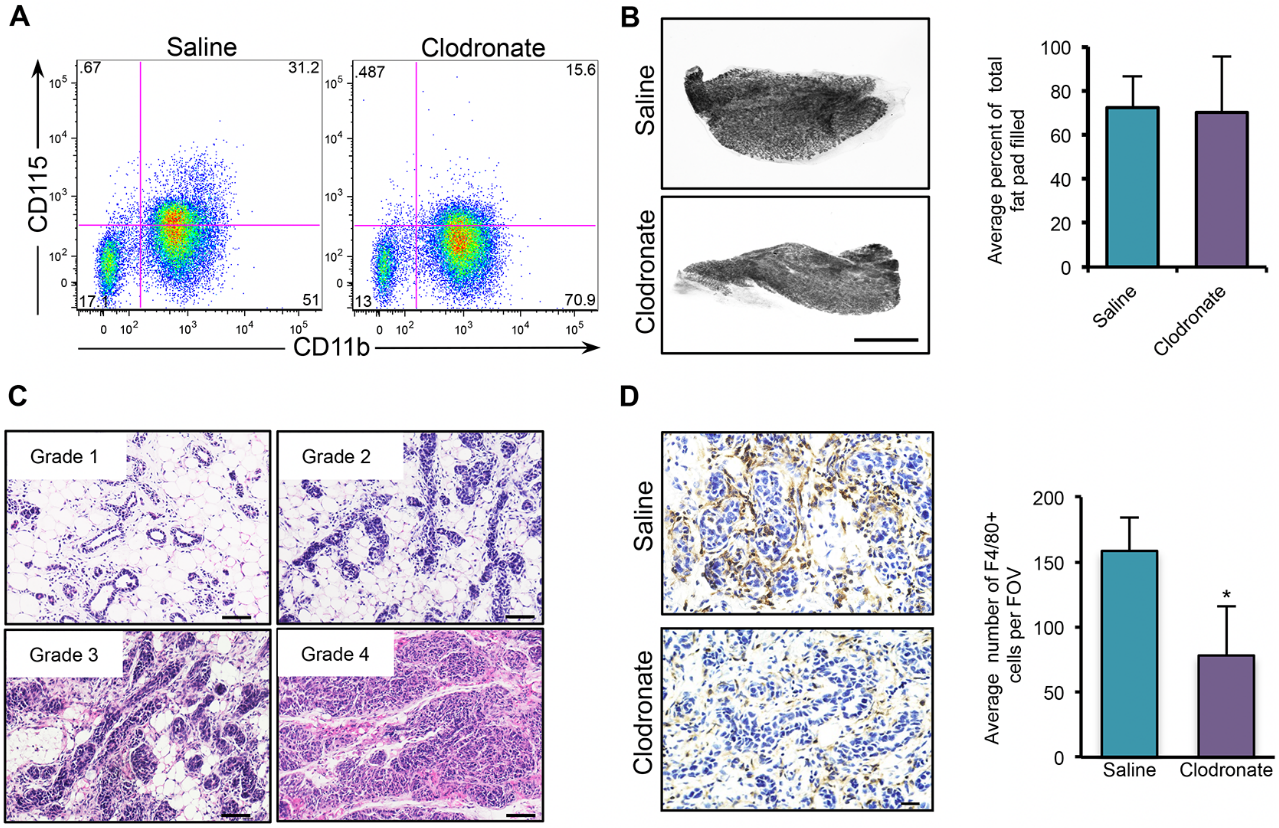

Supplementary Figure 2: Macrophage depletion in PN1a-bearing mice. A. Dot blot depicts the number of $CD11b^{+}CD115^{+}$ cells that were isolated from the bone marrow of saline or clodronate liposome-treated mice bearing PN1a lesions. Cells were gated on SYTOX red^{-} and $CD45^{+}$ subpopulations, and a minimum of 3 mice (3 lesions) were analyzed per a group. **B.** Representative images of carmine-stained saline- and clodronate liposome-treated PN1a lesions. scale bar = 2.5 mm (left). Graph depicts the percent of total fat pad filled where a minimum of 10 lesions (6 mice) were analyzed per group. Values are mean + SD, p=0.79. **C.** H&E staining demonstrating histological grades of PN1a lesions. Grade 1 lesions are characterized by diffuse, well-organized glandular patterns with a single layer of luminal epithelial cells surrounding a central lumen with focal or multifocal hyperplastic regions. Grade 2 lesions have diffuse, well-differentiated hyperplastic ductal and lobuloalveolar patterns, while grade 3 lesions show multifocal regions consisting of solid nests and hyperplastic ductal structures with cytologic atypia. Grade 4 lesions are characterized by solid nests of epithelial cells with little or no glandular differentiation and cytologic atypia. Scale bars = 10 µm. **D.** Representative images of saline- and clodronate liposome-treated lesions stained with an antibody to F4/80 to detect macrophages (left). Graph represents the average number of $F4/80^{+}$ cells per field of view (FOV). Ten FOV were counted for each lesion at 20X magnification, and a minimum of a 10 lesions (6 mice) were analyzed for each group. Values are mean + SD. *p<0.001, Scale bar = 10 µm.

Supplementary Table 1: List of antibodies for immunostaining and flow cytometry

Antibody	Application	Manufacturer	Clone	Dilution
CK8	IF	DSHB	TROMA-1	1:250
CK8	IF	Biolegend	Poly19053	1:200
CK14	IF	Covance	PRB-155P	1:400
CK8	IF	Progen Biotechnik	18.04	1:200
pan-CK	IF	Abcam	AE1/AE3+5D3	1:50
integrin α6	IF	BD Biosciences	GoH3	1:200
Ki67	IF	Abcam	SP6	1:100
Laminin	IF	Sigma	L9393	1:100
F4/80	IHC	AbD Serotec	A3-1	1:100
F4/80	FC	Biolegend	BM8	1:100
MHCII	FC	Biolegend	I-A/I-E	1:100
TIE2	FC	Biolegend	TEK4	1:200
CD3	FC	Biolegend	145-2C11	1:200
CD4	FC	Biolegend	GK1.5	1:200
CD8	FC	Biolegend	53-6.7	1:200
CD11b	FC	eBioscience	M1/70	1:100
CD45	FC	BD Biosciences	30-F11	1:100
CD204	FC	BD Biosciences	2F8	1:100
CD206	FC	Biolegend	C068C2	1:100
CD206	FC	Abcam	EPR6828(B)	1:200

IF: immunofluorescence; IHC: immunohistochemistry; FC: flow cytometry.

Supplementary Table 2: Complete list of differential gene expression in PN1a, PN1b and p53-null mammary glands.

See Supplementary File 1

Supplementary Table 3: Primer sequences for qPCR

Gene symbol	Sense primer (5'-3')	Antisense primer (5'-3')
Il6	AGTCAATTCCAGAAACCGCTATGA	TAGGGAAGGCCGTGGTTGT
Il10	CAGAGCCACATGCTCCTAGA	TGTCCAGCTGGTCCTTTGTT
Il12p40	CAGCCGAGTGATGTACAAGG	TAAACGGGAAATCTGCACCT
Arg1	TTCTCAAAAGGACAGCCTCG	CAGACCGTGGGTTCTTCACA
Nos2	GTCAACTGCAAGAGAACGGAGA	CTGAGAACAGCACAAGGGGTT
Vegfa	AGGCTGCTGTAACGATGAAG	TCTCCTATGTGCTGGCTTTG
Tgfb	TGGAGCAACATGTGGAACTC	GTCAGCAGCCGGTTACCA
Tnfa	CTGTAGCCCACGTCGTAGC	TTGAGATCCATGCCGTTG
18s	GTAACCCGTTGAACCCCATT	CCATCCAATCGGTAGTAGCG
Gas6	GGATTTGCTACCTACAGGCTCA	TTAACTTCCCAGGTGGTTTCC
Gapdh	GCTACACTGAGGACCAGGTTGT	CTCCTGTTATTATGGGGGTCTG

[a] Sequences were designed using the Universal Probe Library Assay Design Center, Roche Applied Biosciences (http://qpcr.probefinder.com/roche3.html)
